# Differentiating the Dilutional Rheology of Radiesse, Radiesse (+), and Radiesse With 0.26 mL of Lidocaine

**DOI:** 10.1111/jocd.16649

**Published:** 2024-10-26

**Authors:** Alec D. McCarthy, Jani van Loghem, Radia El‐Banna, Nadine Hagedorn

**Affiliations:** ^1^ Global Medical Affairs Merz Aesthetics Raleigh North Carolina USA; ^2^ UMA Institute Amsterdam The Netherlands; ^3^ Filler and Medical Device Development Merz Aesthetics GmbH Frankfurt Germany

To the Editor,

The rheological properties of injectable dermal fillers and biostimulators influence their clinical performance, cellular and tissue responses, and adverse event profiles and remain a crucial component in understanding product selection and placement [1, 2, 3]. In our previous publication titled “Dilutional Rheology of Radiesse: Implications for Regeneration and Vascular Safety”, we provide novel insight into the rheological properties of calcium hydroxylapatite‐carboxymethylcellulose (CaHA‐CMC; Radiesse, Merz Aesthetics) gels at various dilutions, revealing that CaHA‐CMC is rheologically sensitive to even small aqueous dilutions [4]. However, these experiments were conducted using only Radiesse classic (CaHA), leading to an influx of inquiries regarding the rheological properties of both Radiesse (+), which contains 0.3% integral lidocaine (CaHA+), and Radiesse classic diluted with 0.26 mL of lidocaine (CaHA Mix Kit), all three of which bear US FDA approvals for injection [5, 6]. Therefore, we have repeated the rheological testing with CaHA(+) and CaHA Mix Kit with measurements taken at 1 Hz using the same methodology and same testing conditions reported in McCarthy et al. and compare the elastic modulus (G′), viscous modulus (G″), and tan(ẟ) with different dilutions of CaHA previously reported [4]. Each of these rheological properties provide insight into both the physical and clinical performance of viscoelastic gels [7]. Their summaries can be found in Table 1.

**TABLE 1:** Rheological measurements, symbols, descriptions, and physico‐clinical correlations reported. Adapted with permission from McCarthy et al. [4].

Measure	Symbol	Rheological description	Physico‐clinical correlate
Elastic (storage) modulus	G′	Ability to store energy elastically and resist shear deformation	*Gel stiffness*: A high G′ equates with a stronger gel that can project tissues and retain its implanted shape
Viscous (loss) modulus	G″	Ability to dissipate energy through shear plastic deformation or flow	*Gel pliability*: A high G′′ indicates the gel is losing strength at an increasing rate, but is meaningful only when compared to G′
Tan delta	Tan *δ* (G″/G′)	Viscoelastic character of a gel based on the ratio of the viscous to elastic modulus (alternatively, the tangent of phase angle *δ*) Colloids with tan *δ* > 1 are more viscous than elastic, behaving more like fluids. Colloids with tan *δ* < 1 are more elastic than viscous, behaving more like solids	*Gel consistency*: Gels with high tan *δ* feel thinner to the touch. In contrast, gels with a low tan *δ* feel thicker or stronger

Summary values G′, G″, and tan(ẟ) are listed in Table 2. CaHA(+)'s G′ is approximately 18% lower than CaHA, but does not reach statistical significance (*p* = 0.0849), while CaHA Mix Kit is approximately 60% lower and does reach statistical significance (*p* < 0.0001) (Figure 1A). The G″ values of CaHA(+) and CaHA Mix Kit are both significantly lower than CaHA (*p* = 0.0003 and *p* < 0.0001, respectively) (Figure 1B). The tan(ẟ) of CaHA(+) is lower than CaHA, but not significantly (*p* = 0.9903) (Figure 1C). A summary of pairwise comparisons between each rheometric property and each CaHA product and dilution are given in the Supporting Information. These values suggest that CaHA(+) retains excellent volumizing properties. Clinically, CaHA(+) in its undiluted form may be exceptionally suited for deep injections and contouring, as the inclusion of lidocaine does not necessitate manual dilution, which is a standard practice when using CaHA as the addition of an FDA‐approved 0.26 mL of lidocaine is generally added to mitigate injection site pain. The findings also suggest that CaHA Mix Kit has significantly lower direct volumization compared to undiluted CaHA and CaHA(+), but compared to other FDA approved hyaluronic acid fillers, would have one of the highest G′ values at 1 Hz [8]. For example, Restylane, Juvederm Voluma, Juvederm Ultra Plus, and Juvederm Ultra have G′ values of approximately 760, 580, 300, and 180 Pa when measured at 1 Hz [9]. Therefore, CaHA, CaHA(+), and CaHA Mix Kit should be regarded as high G′ fillers relative to commercially available HA fillers.

**TABLE 2:** Summary data of the rheological properties from different samples taken at 1 Hz.

Dilution	G′ (Pa)	G″ (Pa)	Tan(*δ*)
CaHA	1310 ± 328.4	584.3 ± 93.07	0.4555 ± 0.04079
CaHA(+)	1075 ± 129.6	476.4 ± 44.27	0.4447 ± 0.0217
CaHA Mix Kit	500.3 ± 9.743	276.9 ± 7.459	0.5537 ± 0.02553
1:0.25	312.5 ± 13.89	193.4 ± 7.18	0.619 ± 0.004583
1:0.5	137 ± 7.146	101.2 ± 2.076	0.7397 ± 0.02715
1:1	38.31 ± 1.838	36.51 ± 0.8968	0.9537 ± 0.02454
1:2	7.538 ± 1.117	10.24 ± 0.8095	1.369 ± 0.1038
1:3	1.824 ± 0.2676	3.579 ± 0.2272	1.977 ± 0.1528

**FIGURE 1:**
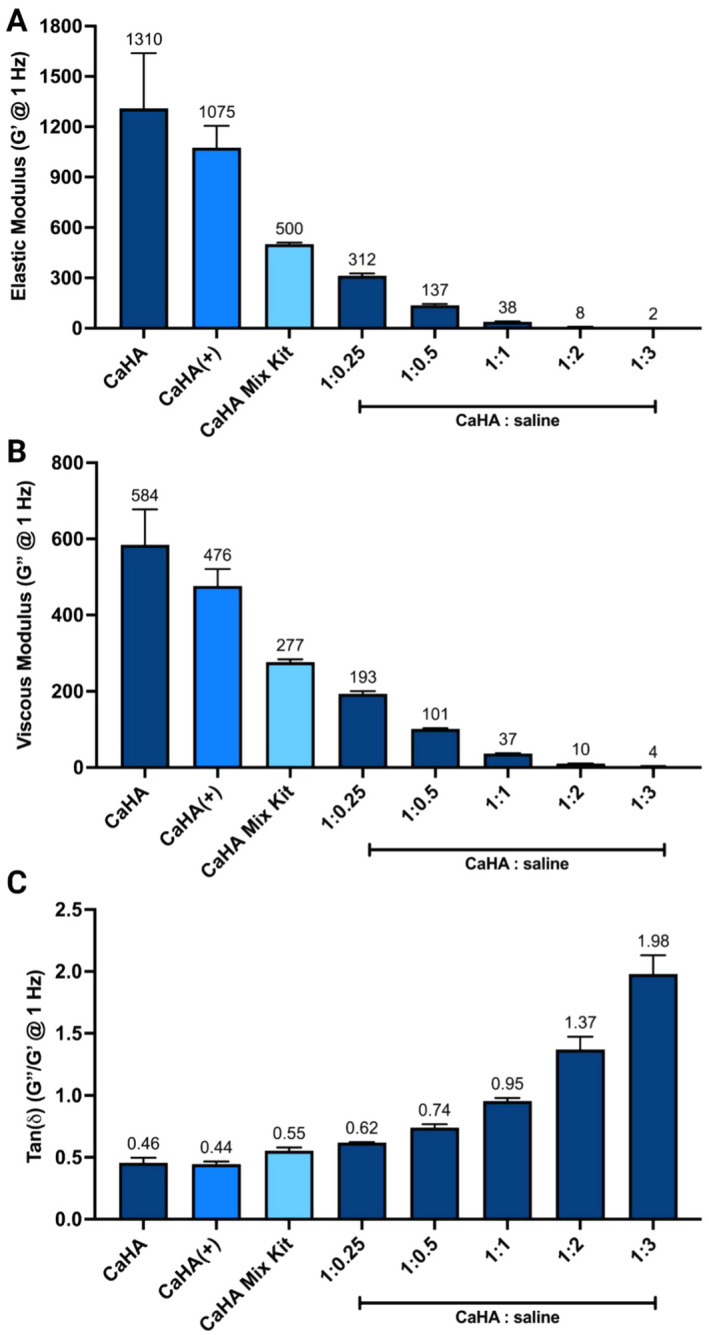
(A) The elastic modulus (G′), (B) viscous modulus (G″), and (C) tan(*δ*) of CaHA at different dilutions, CaHA(+), and CaHA Mix Kit taken at 1 Hz. Adapted from McCarthy et al. [4].

Overall, undiluted CaHA, undiluted CaHA(+), and CaHA Mix Kit possess high G′ values relative to previously reported HA fillers and exhibit predominantly solid‐like behavior, making them uniquely suited for supraperiosteal injections, direct filling, and contouring [10, 11]. Aqueous dilutions of CaHA or CaHA(+) under 1:1 ratios retain direct filling, while dilutions greater than 1:1 function purely through biostimulation. The general understanding of these concepts is reflected in dilution schemes used in clinical practices and in the on‐label dilutions (or lack thereof) by anatomical site, such as a 1:2 dilution for decolletage and undiluted for jawline [5, 12].

## Author Contributions

All authors have made substantial contributions to the conception and design, or acquisition of data, or analysis and interpretation of data, and have been involved in drafting the manuscript or revising it critically for important intellectual content. In addition, all authors have given final approval of the version to be published and agree to be accountable for all aspects of the work.

## Ethics Statement

The authors have nothing to report.

## Conflicts of Interest

Dr. McCarthy, Radia El‐Banna, and Nadine Hagedorn are employed by Merz Aesthetics. Dr. van Loghem is a paid speaker, trainer, and researcher for Merz Aesthetics.

## Supporting information


Data S1.

## Data Availability

The data that support the findings of this study are available from the corresponding author upon reasonable request.
